# Cure of Craving for Stimulants

**Published:** 1874-01

**Authors:** 


The Cure of the Craving for Stimulants.—Dr. Brunton has been writing letters to the *British Messenger* on the temptation to drunkenness caused by the craving for stimulants felt by some people. He furnishes prescriptions which he believes will overcome this craving, and which, we presume, are to be obtained by his readers at the nearest chemists. Here are two of them:

"1st. Put a qarter of an ounce of sulphate of iron and half an ounce of magnesia in an ordinary quart bottle, and fill it with peppermint-water. A wine-glassful to be taken three or four times a day. Instead of the peppermint-water, an infusion of dried peppermint may be used. It may be made stronger or weaker according to the taste of the patient, and should be allowed to cool before it is added to the sulphate of iron and magnesia. A little gum-arabic or gum-tragacanth added to the mixture will keep the magnesia better suspended, but this may perfectly well be omitted. The bottle should be shaken before the dose is poured out. 2d. Take an ounce of quassia chips and pour over them as much cold water as will fill three quart bottles. Let them stand an hour, and then strain. Add to the strained liquid six and a half fluid drachms of the solution of iron, sold under the name of 'Liquor Ferri Perchloridi.' Two tablespoonfuls or half a wine-glassful to be taken three or four times a day. The iron solution may be measured out with a teaspoon, one teaspoonful being equal to one fluid drachm; but teaspoons vary in size, and it is therefore better to use a glass measure, which may be bought at any chemist's."

No doubt there are many cases in which a chalybeate is indicated, but it may be questioned whether it would not be wiser of those who wish to try the plan to ask a medical man first. The value of such advice is indicated by the following remarks appended by Dr. Brunton to the recipes we have quoted:

"When the person's tongue is pale, flabby and marked with teeth at the edges, the second prescription may be found more useful than the first. When there is any tendency to flatulence the first should be taken a quarter of an hour before meals; and if either of them causes uneasiness immediately after meals.

"In persons of a robust habit or florid complexion, the following prescription, which I owe to the kindness of Mr. John Groom, of Hempstead, may be found more serviceable than either of the preceding. Add one ounce of bruised gentian root to one quart of boiling water. Let this stand four hours; then strain off the liquor, and add two drachms of carbonate of ammonia.

A wine-glassful may be taken two or three times a day when the craving comes on.

"This prescription was used by Mr. Fox (now of Brighton) when surgeon to Bedford Jail. Though I have recommended it in certain cases in preference to the other prescription, it may be used by all who are addicted to the use of intoxicating drinks." *The Clinic.*

